# Structural proteomics of minimal organisms: Conservation of protein fold usage and evolutionary implications

**DOI:** 10.1186/1472-6807-6-7

**Published:** 2006-03-28

**Authors:** John-Marc Chandonia, Sung-Hou Kim

**Affiliations:** 1 Physical Biosciences Division, Lawrence Berkeley National Laboratory, Berkeley, CA 94720, USA; 2 Department of Chemistry, University of California, Berkeley, CA 94720, USA

## Abstract

**Background:**

Determining the complete repertoire of protein structures for all soluble, globular proteins in a single organism has been one of the major goals of several structural genomics projects in recent years.

**Results:**

We report that this goal has nearly been reached for several "minimal organisms" – parasites or symbionts with reduced genomes – for which over 95% of the soluble, globular proteins may now be assigned folds, overall 3-D backbone structures. We analyze the structures of these proteins as they relate to cellular functions, and compare conservation of fold usage between functional categories. We also compare patterns in the conservation of folds among minimal organisms and those observed between minimal organisms and other bacteria.

**Conclusion:**

We find that proteins performing essential cellular functions closely related to transcription and translation exhibit a higher degree of conservation in fold usage than proteins in other functional categories. Folds related to transcription and translation functional categories were also overrepresented in minimal organisms compared to other bacteria.

## Background

The availability of complete genome sequences opened up a new era in biology, providing a global and systems view of the range of genome sizes in different organisms, the presence or absence of genes involved in various cellular functions, the genes involved in particular cellular functions, and the relative abundance of different gene families. This new global view is creating major new areas of research such as functional genomics [1]. At the time of this writing, over 224 prokaryotic genomes and over 22 complete eukaryotic genomes have been sequenced [2]. Just as the field of sequence genomics has yielded complete genome sequences for a variety of organisms, the field of structural genomics aims to provide structures for the complete array of biological macromolecules found in nature, [3-7]. The first phase of structural genomics focused only on proteins (not RNAs), and has proven to be an efficient means of providing structural information for new protein families [8-10].

After the first sequencing of a complete genome of *Haemophilus influenzae *[11], some of the earliest subsequent genomes sequenced were from the "minimal organisms" *Mycoplasma genitalium *and *M. pneumoniae *[12,13]. Minimal organisms have been the subject of numerous experimental and computational genomic studies because of the possibility of identifying the minimal complement of genes necessary for sustaining life [14-16]. Because of their small size, organisms with minimal genomes have also been popular for structure and function prediction [13,17-24]. The minimal organisms *M. genitalium *(~486 protein-encoding genes) and *M. pneumoniae *(~690 genes) have also been the focus of structural genomics research at the Berkeley Structural Genomics Center [25,26].

Other minimal organisms that have been sequenced more recently include the aphid symbiont *Buchnera aphidicola *(~572 genes) [27], the ant symbiont *Candidatus Blochmannia floridanus *(~583 genes) [28], the tsetse fly symbiont *Wigglesworthia glossinidia brevipalpis *(~612 genes) [29], and the Whipple's disease parasite *Tropheryma whipplei *(~781 genes) [30]. Comparative analysis of the first three symbiont genomes and *M. genitalium *has demonstrated that the symbionts are closely related, sharing 313 orthologous genes (51–55% of each genome), and that they share 179 genes with *M. genitalium *[31]. However, a broader comparison of all five species, including *T. whipplei*, indicated significant variability in the functional repertoire of proteins in these organisms, suggesting that minimal genomes are not the result of a unique reductive evolutionary pathway, but the products of reductive evolution in specific environments [32].

A recent survey of proteins from 238 complete genomes revealed that fold assignments (approximate 3-D backbone structures) can be made for the majority of non-membrane proteins of minimal organisms [33]. Statistically significant sequence similarity to a protein of known structure allows homology (evolutionary relatedness) to be inferred, thus enabling the fold of the homologous proteins to be assigned even in cases where the degree of sequence similarity is insufficiently high to allow accurate modeling [34].

Fold assignment of a protein has implications for functional annotation, because the link between molecular function and structure is well known. Todd and colleagues showed that while the majority of superfamilies display variation in enzyme function (i.e., molecular function), the biochemical mechanisms (as represented by the Enzyme Commission [EC] number) are almost always conserved between proteins with 40% sequence identity or above [35]. More recent work has shown that conserved domain combinations, or supradomains, are more likely to maintain a conserved molecular function even at lower sequence identity [36]. A study in two proteomes (yeast and *Escherichia coli*) found clear tendencies for fold-function association across a broad range of molecular functions [37]. The latter study also found the fold distributions in the two proteomes surveyed did not vary significantly from the average across all sequenced proteomes, although the study was based on fold assignments for less than 10% of the total number of proteins.

We now report that recent efforts in structural biology and structural genomics have succeeded in enabling fold assignments for over ~90% of soluble, globular proteins in the five minimal organisms described above. In this report, we survey the classes of protein folds found in each organism, and examine the conservation in fold usage of proteins in several broad categories of cellular function. We find that the degree of conservation of fold usage varies among cellular functional categories, with the most conserved categories of proteins performing essential cellular functions closely related to transcription and translation. Finally, we compare the degree of conservation in cellular functions and fold usage among the five minimal organisms and *E. coli*, a non-minimal organism.

## Results and discussion

### Near-complete coverage of soluble, globular proteomes of "minimal" organisms

In Table 1, we show the percentage of proteomes that may be assigned folds for five minimal organisms and for *E. coli*, an example of a well-studied organism that is not "minimal." For the minimal organisms considered in this study, nearly all proteins annotated as soluble and globular may be assigned to a known fold. The aphid symbiont *B. floridanus *has the highest coverage, at 96% of soluble, globular proteins (431 of 451 proteins). 58 of the remaining proteins in the proteome (10% of the proteome) have unknown structure, but are predicted to have at least one transmembrane helix. 3 additional proteins have unknown structure and no predicted transmembrane helices, but 20% or more of their residues are in predicted low complexity or coiled coil regions, and thus not easily tractable in experimental structural studies. Overall, the folds of 502 of 583 *B. floridanus *proteins (86%) may be annotated by sequence similarity to a protein of known structure. Other minimal organisms also have high structural coverage: 95% of soluble, globular *W. glossinidia *proteins, 94% of soluble, globular *B. aphidicola *proteins, 87% of soluble, globular *M. genitalium *proteins, and 87% of soluble, globular *T. whipplei *proteins can reliably be assigned folds. In contrast, only 78% of soluble, globular *E. coli *proteins can reliably be assigned folds. The low numbers of predicted transmembrane proteins in several of the minimal organisms (e.g., only 87 of 572 *B. aphidicola *proteins) is also notable; previous analyses suggest that some transmembrane proteins (e.g., proteins with a role in cell defense or transporters of diverse nutritional sources) are less important to intracellular symbiotes than to free-living bacteria [27].

### α/β fold class is the most common category of fold

**Figure 1:**
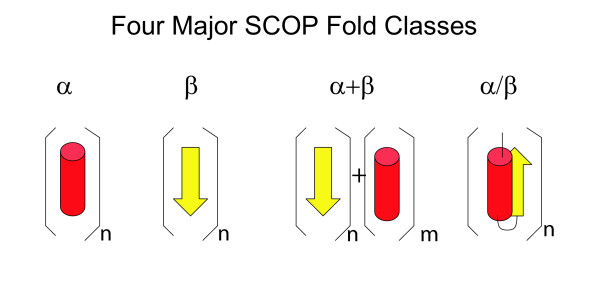
**Four major SCOP classes**. The predominant form of secondary structure in each of the first four SCOP classes is shown. Alpha helices are shown as red cylinders, and beta strands as yellow ribbons.

For the proteins that could be reliably assigned folds, we examined their structural classification in the SCOP database [38]. SCOP is a widely used, manually curated database in which protein structures are divided into domains, which are classified in a hierarchy indicating different types of structural and evolutionary relationships between the domains. Domains classified together in a single "family" or "superfamily" are hypothesized to have a common evolutionary origin on the basis of sequence or structural evidence. Superfamilies that share similar secondary structural features and topology, but for which there is little or no evidence to suggest a common evolutionary origin, are classified together at the "fold" level. SCOP folds are grouped together in seven major "classes" (all-α, all-β, α/β, α+β, multi-domain, membrane, and small), based on common physical characteristics such as the predominant type of secondary structure or the order of connection of the different secondary structures (Figure 1). Note that the SCOP "multi-domain" class encompasses folds that are comprised of multiple domains that individually would belong to different classes; individual domains from multi-domain proteins are not classified in the "multi-domain" class. Although we use the term "fold" to refer to a protein's overall 3D backbone structure, we use the term "SCOP fold" to refer to a specific fold classification within the SCOP database.

**Figure 2:**
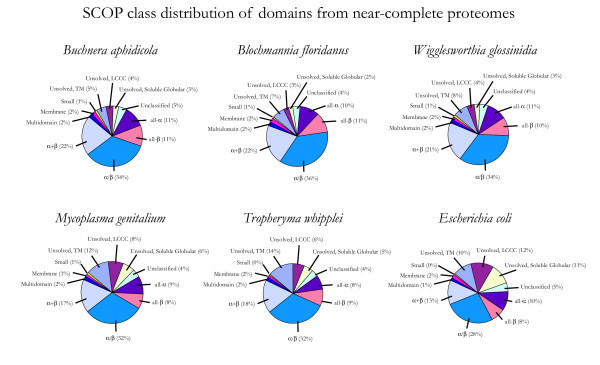
**SCOP class distribution in near-complete proteomes**. The fraction of domains in each proteome belonging to each of the first 7 SCOP classes is shown. "Unclassified" domains are from proteins annotated as homologous to a known structure using Pfam, but not classified in one of the first 7 classes of SCOP (e.g., due to being in a superfamily solved since the SCOP cutoff date of 15 May 2004). "Unsolved" domains are from proteins not annotated as homologous to a known structure. For statistical analysis, each ORF in the latter two categories was treated as containing exactly one domain. "Unsolved" domains are further divided into three categories based on predicted tractability in high-throughput experiments: "Unsolved, TM" are predicted to contain at least one transmembrane helix, "Unsolved, LCCC" have no predicted transmembrane helices but at least 20% of the sequence in low complexity or coiled coil regions, and "Unsolved, Soluble Globular" are predicted to be tractable in high-throughput experiments due to having neither of these features.

The fraction of proteins found in each organism belonging to each of these SCOP classes is shown in Figure 2. Those proteins that could not reliably be assigned folds, and those that were assigned a fold based on homology to a protein not yet classified in SCOP, are described as "Unsolved" and "Unclassified," respectively. For all organisms, the highest proportion of SCOP folds are in the α/β class, and those in the α/β and α+β classes together comprise over half of the assigned SCOP folds. This reflects the observation that the α/β class contains some of the most functionally diverse "superfolds" that act as scaffolds for a wide array of molecular or chemical functions [39].

### Usage of protein fold classes are conserved for key cellular processes

In order to analyze how the annotated cellular function of each protein correlates with its structure, we examined the "functional role" annotation for each protein as provided in the TIGR database [40]. We found that the distribution of proteins among SCOP fold classes was highly conserved within some roles and showed much more variability in others.

**Figure 3:**
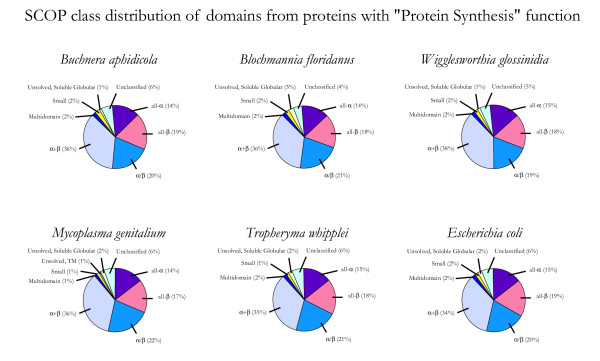
**SCOP class distribution of proteins with "Protein Synthesis" function**. The fraction of domains in each proteome from the TIGR role category "Protein Synthesis" belonging to each of the first 7 SCOP classes is shown. "Unclassified" and "Unsolved" domains were counted as described in Figure 1.

Figure 3 shows the fold class distribution of proteins in the "Protein Synthesis" functional category across all 6 proteomes. The fraction of these proteins in each structural class shows little variability, with no more than a 4% difference between proteomes. Furthermore, the proteins in this functional category comprise a relatively large fraction of the proteins in each proteome (99 proteins on average, or 8% of the proteome). The extremely low variability is consistent with the idea that these proteins have been fundamental part of cellular biochemistry since early evolution, and are thus essential to any organism regardless of its environment.

**Figure 4:**
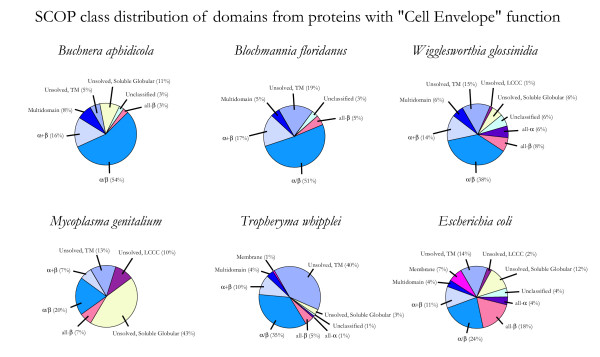
**SCOP class distribution of proteins with "Cell Envelope" function**. The fraction of domains in each proteome from the TIGR role category "Cell Envelope" belonging to each of the first 7 SCOP classes is shown. "Unclassified" and "Unsolved" domains were counted as described in Figure 1.

In contrast, Figure 4 shows the fold class distribution of proteins in the "Cell Envelope" functional category across all 6 proteomes. This functional category is also highly represented in each proteome (73.8 proteins on average), but the proteins show a much higher degree of variation in fold usage. This category contains the highest proportion of unassigned folds, as well as a diverse array of assigned SCOP folds: for example, 6% and 4% of domains from *W. glossinidia *and *E. coli *cell envelope proteins belong to the all-α structural class, while cell envelope proteins from the other proteomes contain few or no all-α structures. *E. coli *also contains a number of solved transmembrane structures, while other proteomes contain significant numbers of proteins predicted to be transmembrane proteins not detectably homologous to any protein with a known 3D structure. *M. genitalium *and *T. whipplei *contain the largest fractions of cell envelope proteins that could not be reliably assigned a fold at this time, although most of these *M. genitalium *proteins are expected to be soluble and globular, while the majority of such proteins from *T. whipplei *are predicted to contain at least one transmembrane helix. The high amount of variability suggests that proteins in the "Cell Envelope" category evolve rapidly in response to specific pressures in an organism's environment, and different sets of these proteins remain after reductive evolution in the different environments occupied by the different species of minimal organisms.

### Cellular functions with most conserved SCOP fold usage

Previous comparative sequence genomic analyses of symbionts have shown that the number of proteins in most cellular function categories varies little between symbiont proteomes, and that many of the most highly conserved proteins have cellular functions related to information storage and processing, particularly translation and ribosomal structure [31]. We calculated the coefficient of variation (CV) in the number of proteins in each functional role category (N_1 _for the first species, N_2 _for the second species, etc.), as shown in Equation 1.

CVsequence=Stdev(N1…N6)Mean(N1…N6)     (1)
 MathType@MTEF@5@5@+=feaafiart1ev1aaatCvAUfKttLearuWrP9MDH5MBPbIqV92AaeXatLxBI9gBaebbnrfifHhDYfgasaacH8akY=wiFfYdH8Gipec8Eeeu0xXdbba9frFj0=OqFfea0dXdd9vqai=hGuQ8kuc9pgc9s8qqaq=dirpe0xb9q8qiLsFr0=vr0=vr0dc8meaabaqaciaacaGaaeqabaqabeGadaaakeaacqWGdbWqcqWGwbGvdaWgaaWcbaGaem4CamNaemyzauMaemyCaeNaemyDauNaemyzauMaemOBa4Maem4yamMaemyzaugabeaakiabg2da9maalaaabaGaem4uamLaemiDaqNaemizaqMaemyzauMaemODayNaeiikaGIaemOta40aaSbaaSqaaiabigdaXaqabaGccqWIMaYscqWGobGtdaWgaaWcbaGaeGOnaydabeaakiabcMcaPaqaaiabd2eanjabdwgaLjabdggaHjabd6gaUjabcIcaOiabd6eaonaaBaaaleaacqaIXaqmaeqaaOGaeSOjGSKaemOta40aaSbaaSqaaiabiAda2aqabaGccqGGPaqkaaGaaCzcaiaaxMaadaqadaqaaiabigdaXaGaayjkaiaawMcaaaaa@59BA@

Results are shown in Table 2. As expected, the category with the lowest variation in the number of proteins is "Protein synthesis," and the top three categories are all closely related to transcription or translation.

We also calculated the coefficient of variation in the number of protein domains assigned to each SCOP class (N_1,all-α _for the first species in the all-α class, N_2,all-α _for the second species in the all-α class, etc), then averaged that data across all 7 structural classes, as shown in Equation 2.

CVstructure=∑class=17stdev(N1,class…N6,class)Mean(N1,class…N6,class)7     (2)
 MathType@MTEF@5@5@+=feaafiart1ev1aaatCvAUfKttLearuWrP9MDH5MBPbIqV92AaeXatLxBI9gBaebbnrfifHhDYfgasaacH8akY=wiFfYdH8Gipec8Eeeu0xXdbba9frFj0=OqFfea0dXdd9vqai=hGuQ8kuc9pgc9s8qqaq=dirpe0xb9q8qiLsFr0=vr0=vr0dc8meaabaqaciaacaGaaeqabaqabeGadaaakeaacqWGdbWqcqWGwbGvdaWgaaWcbaGaem4CamNaemiDaqNaemOCaiNaemyDauNaem4yamMaemiDaqNaemyDauNaemOCaiNaemyzaugabeaakiabg2da9maalaaabaWaaabmaeaadaWcaaqaaiabdohaZjabdsha0jabdsgaKjabdwgaLjabdAha2jabcIcaOiabd6eaonaaBaaaleaacqaIXaqmcqGGSaalcqWGJbWycqWGSbaBcqWGHbqycqWGZbWCcqWGZbWCaeqaaOGaeSOjGSKaemOta40aaSbaaSqaaiabiAda2iabcYcaSiabdogaJjabdYgaSjabdggaHjabdohaZjabdohaZbqabaGccqGGPaqkaeaacqWGnbqtcqWGLbqzcqWGHbqycqWGUbGBcqGGOaakcqWGobGtdaWgaaWcbaGaeGymaeJaeiilaWIaem4yamMaemiBaWMaemyyaeMaem4CamNaem4CamhabeaakiablAciljabd6eaonaaBaaaleaacqaI2aGncqGGSaalcqWGJbWycqWGSbaBcqWGHbqycqWGZbWCcqWGZbWCaeqaaOGaeiykaKcaaaWcbaGaem4yamMaemiBaWMaemyyaeMaem4CamNaem4CamNaeyypa0JaeGymaedabaGaeG4naCdaniabggHiLdaakeaacqaI3aWnaaGaaCzcaiaaxMaadaqadaqaaiabikdaYaGaayjkaiaawMcaaaaa@877C@

**Figure 5:**
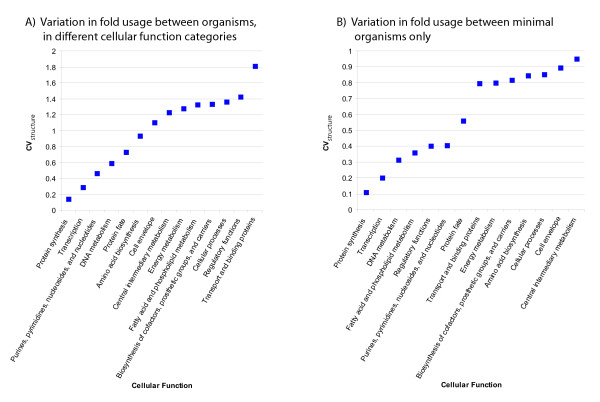
**Variation in fold usage between organisms differs between functional categories**. A) Variation in fold usage (CV_structure_) between organisms within each TIGR role category is shown for each category that represents a cellular function. The data are also given in the "fold-based variation" column in Table 2. B) Variation in fold usage between minimal organisms only, excluding *E. coli *data as per Table 3.

CV_structure _was calculated separately for each functional role category, and these data are shown in Table 2 and Figure 5A. The functional category with the lowest variation in the number of domains in each structural class is "Protein Synthesis," as would be expected from Figure 3. However, there are some interesting differences between the rankings based only on the CV_sequence_, and the rankings based on CV_structure_. For example, fold usage of proteins involved in biosynthesis of cofactors, carriers, and prosthetic groups varies to a higher degree than the variation in total numbers of these proteins in each proteome. This implies that the repertoire of specific functions in this broad category is specialized to the particular needs of each organism, even though the overall number of such proteins varies little. As expected, the distribution of structures in "catch-all" classes such as hypothetical and unclassified proteins are more varied than the distribution of structures found in more well-defined functional categories.

We also analyzed the degree of variation using data from only the five near-complete minimal organisms, excluding data from *E. coli*. Results are shown in Table 3 and Figure 5B. As before, fold usage of proteins in the "protein synthesis" category shows the least variance of all functional categories. The total genome size also slows relatively little variation among minimal organisms, as has been observed previously [31]. However, some functional categories show relatively more variation between minimal organisms than between minimal organisms and *E. coli*. For example, the cellular function categories "Cell envelope," "Central intermediary metabolism," and "Amino Acid Biosynthesis" all drop in rank (the relative degree of conservation in fold usage among functional categories) by 7 positions relative to Table 2, indicating higher diversity of folds in these functional categories among minimal organisms. In contrast, fold usage of proteins in the "Regulatory functions" category shows relatively less variation among minimal organisms than between minimal organisms and *E. coli*. This suggests that although the minimal organisms have lost many of the regulatory pathways unnecessary for survival in their relatively unchanging environments, they maintain a relatively conserved set of proteins responsible for common regulatory functions. A more thorough phylogenetic analysis of these proteins would be necessary to test this hypothesis.

### Common and overrepresented folds in minimal organisms

We examined the most common protein folds (as defined in SCOP 1.67) in minimal organisms. Results are shown in Table 4. Four of the eleven most common SCOP folds (TIM barrel, nucleoside triphosphate hydrolase, flavodoxin-like, and ferredoxin-like) are among the nine superfolds originally described by Orengo and colleagues as scaffolds that can support a wide array of molecular functions [39]. However, all have fewer copies in minimal organisms than are found in *E. coli*.

Table 5 shows SCOP folds that are found in both minimal organisms and in *E. coli*, which are represented at equal or greater levels in the minimal organisms. Proteins with these folds are presumably important for the survival of the organisms, and were not eliminated during reductive evolution. Five SCOP folds are present in slightly greater numbers in minimal organisms than in *E. coli*. For example, the DNA primase core fold (e.13) has 3 representatives in *M. genitalium*: the DNA primase protein itself (dnaE) and two conserved hypothetical proteins (NP_072670 and NP_072719). All five folds are involved in the critical functions of transcription, translation, or DNA replication. Forty-two other SCOP folds are present in the same numbers in each minimal genome as in *E. coli*. The five with the largest number of copies per genome are shown in Table 5. Some appear to be key metabolic enzymes, while others are involved in transcription, translation, or DNA replication.

Interestingly, all 47 SCOP folds present in equal or greater numbers in all minimal organisms as in *E. coli *are also folds for which only a single superfamily is characterized in SCOP; i.e., all proteins sharing the fold are also annotated as evolutionarily related to each other. The case of multiple superfamilies sharing one fold may arise from two alternative causes: convergent evolution of two or more families to one fold, or a single family that has diverged enough that homology between different branches of the family are no longer evident even from structure (in this case, each branch would be classified as a different superfamily in SCOP). These data imply that proteins that play sufficiently important roles to avoid elimination during reductive evolution have also not diverged as much as other protein families due to this same evolutionary pressure.

An additional set of SCOP folds found only in minimal organisms and not in *E. coli *is given in Table 6. None of these folds are found in all five minimal organisms, and the proteins are not generally related to essential cellular functions such as transcription, translation, or replication. Some are presumably adaptations to the specific environment of the organism, and several (e.g., viral coat and capsid proteins, and the MHC antigen-recognition domain) are not typically found in bacteria. These may represent lateral gene transfers or erroneous annotations.

## Conclusion

After five years of progress in structural genomics, near-complete structural complements of the soluble proteins of several "minimal organisms" are now known. A complete set of fold assignments for nearly all soluble, globular proteins in a proteome is providing a global view of how minimal organisms are using various protein fold classes for different cellular functions and how the fold usage in each class is conserved.

Data from near-complete structural proteomes can yield hypotheses on protein evolution at a global level. Simple statistical analyses of the variation in numbers of structures in each structural and functional category can shed light on which functional categories are more or less conserved in minimal organisms. For example, the functional categories that showed the least variability in both sequence- and structure-based analyses were involved in essential cellular functions such as transcription and translation. Furthermore, every SCOP fold identified in equal or greater numbers in minimal organisms as in *E. coli *was the product of a single protein family, indicating that the proteins retained during reductive evolution of minimal organisms also tend to be from slow-evolving families. The latter observation was expected, as essential genes in other species have previously been shown to evolve more slowly than non-essential genes [41,42].

Such observations may be followed up with more detailed studies based on phylogenetic modeling of protein families [43] or the construction of atomic models of proteins in those categories. Detailed atomic modeling of all proteins in a biochemical pathway will be useful to study the plasticity of these pathways in response to evolutionary pressures imposed by different organisms' environments [44].

## Methods

### Databases

Our database of known protein structures, knownstr, was created on 22 Feb 2005. This database contained sequences of every protein chain released by the PDB [45], including those of obsolete entries, sequences of proteins deposited in the PDB and made available while the structures were still on hold, and sequences from TargetDB [46], for which a structure had been solved by a participating structural genomics center.

Pfam [47] classification of known structures was evaluated using Pfam version 16.0. The HMMER tool (version 2.3.2) [48] was used to compare the Pfam_ls library of hidden Markov models to the knownstr database, using the family-specific "trusted cutoff" score as a cutoff for assigning significance.

INTEGR8 version 12 [2] was used for sequence data. The Integr8 database contains data for 238 complete proteomes, including 19 eukaryotes. The proteome for each organism is composed of proteins curated from the Swiss-Prot and TrEMBL databases. All proteins were annotated with hidden Markov models [48,49] from the InterPro [50] database. Since InterPro includes models from Pfam, we used the supplied InterPro annotations to map Pfam domains onto each protein. The version of InterPro used to annotate Integr8 version 12 includes Pfam 16.0

SUPERFAMILY [51] version 1.67 contains hidden Markov models based on superfamilies from the SCOP database [38,52], also version 1.67. Recent versions of SUPERFAMILY [53] provide pre-calculated annotations of genomes downloaded from NCBI with all the superfamily models. We used these precalculated annotations to assign SCOP domains to sequences from minimal organisms and *E. coli*, as described below. The false positive rate for SUPERFAMILY annotations is estimated to be less than 1% [54].

The Comprehensive Microbial Resource [40] contains annotations of TIGR role categories in its OMNIOME database. We obtained TIGR role annotations from the version of OMNIOME downloaded on 12 May 2005. Of 19 TIGR role categories, two ("signal transduction" and "other categories") were found in low average abundance in the proteomes we analyzed (averaging 0.7 and 9.0 proteins per proteome, respectively), and these categories were excluded from our analysis. The remaining 17 categories are listed in Table 2.

### Mapping annotations

To use annotations from the SUPERFAMILY and OMNIOME databases, we mapped proteins from the Integr8 database onto corresponding proteins in the NCBI and CMR Locus databases, respectively. In most cases, this was done by mapping identical sequences from the corresponding genome. However, in some cases, the gene or ORF annotations of the same genomes varied between the databases, resulting in different protein sequences. In these cases, we used BLAST [55] version 2.2.9 to map each Integr8 sequence to the most similar sequence in the other databases. We mapped each protein in Integr8 that could not be mapped by direct sequence match to the most significant BLAST hit in the other database, provided the BLAST E-value of the hit at least as significant as an empirically chosen threshold of 10^-10^. An average of 16.3 proteins in each proteome could not be mapped to any of the functional categories in OMNIOME, and were not included in this analysis.

### Predicting tractability in high-throughput experiments

We identified all proteins with a predicted transmembrane helix, or with 20% or more residues in low complexity regions, or with 20% or more residues in coiled coil regions, as likely to be intractable in high-throughput experiments. Other proteins were annotated as soluble, globular proteins. The 20% threshold were used in more recent target selection rounds at the Berkeley Structural Genomics Center [25]. Similar thresholds have also been justified by recent comprehensive crystallization trials on the *Thermotoga maritima *proteome [56].

The "seg" program [57] (version dated 5/24/2000) was run on all sequences in Integr8 to identify putative low complexity regions. The "ccp" program [58] (version dated 6/14/1998) was used to predict coiled coil regions in all sequences, and TMHMM 2.0a [59] was used to predict the locations of transmembrane helices. TMHMM can distinguish between soluble and membrane proteins with both specificity and sensitivity greater than 99%, but frequently produces false positive predictions when signal peptides are present. Default options were used for all programs.

## Authors' contributions

JMC designed the study, carried out the analyses, and drafted the manuscript. SHK and JMC jointly made conceptual design of the study and interpreted the results, and SHK helped draft the manuscript. Both authors read and approved the final manuscript.

## Figures and Tables

**Table 1:** Status of near-complete structural proteomes as of 22 February 2005. How many proteins may be assigned folds in near-complete proteomes? The status for five near-complete prokaryotes are shown. *E. coli*, a well-studied bacteria that is not considered a minimal organism, is included for comparison.

**Organism**	**Total # of proteins**	**# of soluble, globular proteins**	**# of soluble, non-globular proteins**	**# of membrane proteins**	**# of folds assigned**	**% folds assigned (of total)**	**% folds assigned(of soluble, globular)**	**# of remaining soluble, globular proteins**	**# of remaining soluble, non-globular proteins**	**# of remaining membrane proteins**
*Candidatus Blochmannia floridanus*	583	451	12	120	502	86.1%	95.6%	20	3	58
*Wigglesworthia glossinidia brevipalpis*	612	536	28	217	508	83.0%	94.8%	28	7	69
*Buchnera aphidicola (subsp. Acyrthosiphon pisum)*	572	446	39	87	495	86.5%	94.4%	25	9	43
*Mycoplasma genitalium*	486	341	34	111	350	72.0%	87.1%	44	10	82
*Tropheryma whipplei (strain TW08/27)*	781	430	55	127	556	71.2%	87.0%	56	15	154
*Escherichia coli*	4338	3130	146	1062	2945	67.9%	78.0%	688	76	629

**Table 2:** Variation within functional categories based on sequence and structure. Which functional categories show the most variation in fold usage between organisms? The first column lists 17 TIGR cellular function categories, and an additional category composed of all proteins in each proteome. The "fold-based variation" column is based on a calculation of the coefficient of variation in the number of structurally characterized domains in each functional role in each of the first 7 SCOP classes (all-α, all-β, α/β, α+β, multi- domain, membrane, small). As described in Equation 2, the coefficient of variation is calculated separately for each of the 7 classes, and then averaged across all 7 classes to produce CV_structure_. The "sequence-based variation" column gives the coefficient of variation in the number of proteins in each category (CV_sequence_, Equation 1). The "fold-based rank" and "sequenced-based rank" show the ranking of functional categories based on the amount of fold-based and sequence-based variation, from lowest amount of variation to the highest. Cellular function categories are ordered in the table according to their fold-based rank.

**Category**	**Average # of Proteins**	**Fold-based variation (CV_structure_)**	**Sequence-based variation (CV_sequence_)**	**Fold-based Rank/Sequence-based Rank**
Protein synthesis	99.0	0.141	0.100	1/1
Transcription	20.8	0.286	0.409	2/2
Purines, pyrimidines, nucleosides, and nucleotides	36.8	0.462	0.570	3/3
DNA metabolism	46.8	0.586	0.753	4/6
Protein fate	48.3	0.731	0.723	5/4
Amino acid biosynthesis	44.7	0.935	0.972	6/8
**All Proteins**	1228.7	1.061	1.242	7/12
Cell envelope	73.8	1.099	0.971	8/7
Central intermediary metabolism	27.5	1.228	1.113	9/10
Energy metabolism	116.7	1.276	1.220	10/11
Fatty acid and phospholipid metabolism	20.0	1.328	1.014	11/9
Biosynthesis of cofactors, prosthetic groups, and carriers	50.3	1.332	0.731	12/5
Cellular processes	62.0	1.364	1.301	13/13
Regulatory functions	34.5	1.427	1.940	14/18
Unknown function	115.6	1.659	1.865	15/17
Transport and binding proteins	81.8	1.809	1.638	16/15
Hypothetical proteins	205.8	1.984	1.631	17/14
Unclassified	118.0	2.020	1.835	18/16

**Table 3:** Variation within functional categories in minimal organisms. Which functional categories show the most variation in fold usage between minimal organisms? The data are calculated as in Table 2, but ignore data from *E. coli*. The structure-based variation when *E coli *data are included (from Table 2) is provided for comparison.

**Category**	**Average # of Proteins**	**Fold-based variation (CV_structure_)**	**Fold-based variation, including *E. coli***	**Sequence-based variation(CV_sequence_)**	**Fold-based Rank/Sequence-based Rank**
Protein synthesis	95.2	0.108	0.141	0.039	1/1
Transcription	17.6	0.200	0.286	0.199	2/3
**All Proteins**	606.8	0.210	1.061	0.178	3/2
DNA metabolism	33.0	0.314	0.586	0.328	4/6
Fatty acid and phospholipid metabolism	12.0	0.358	1.328	0.486	5/9
Regulatory functions	7.2	0.402	1.427	0.465	6/8
Purines, pyrimidines, nucleosides, and nucleotides	28.8	0.405	0.462	0.284	7/4
Protein fate	34.6	0.560	0.731	0.303	8/5
Unknown function	27.8	0.776	1.659	0.555	9/12
Transport and binding proteins	27.4	0.796	1.809	0.574	10/13
Energy metabolism	59.4	0.799	1.276	0.454	11/7
Biosynthesis of cofactors, prosthetic groups, and carriers	38.6	0.816	1.332	0.666	12/15
Amino acid biosynthesis	29.0	0.844	0.935	0.782	13/17
Cellular processes	29.8	0.853	1.364	0.636	14/14
Cell envelope	45.8	0.893	1.099	0.506	15/10
Central intermediary metabolism	15.4	0.952	1.228	0.552	16/11
Unclassified	30.0	1.006	2.020	0.749	17/16
Hypothetical proteins	70.6	1.125	1.984	0.871	18/18

**Table 4:** Most common SCOP folds in minimal organisms. Which SCOP folds are most common in minimal organisms? The first column gives the name and SCOP sccs identifier for folds classified in SCOP 1.67. The second column gives the total number of domains assigned to each fold among the five minimal organisms. The third column is calculated as the average number of domains among the five minimal organisms studied that were assigned to each fold, divided by the number of domains in *E. coli *assigned to the same fold.

**Fold Name**	**Number**	**Ratio**
P-loop containing nucleoside triphosphate hydrolases (c.37)	319	0.23
TIM beta/alpha-barrel (c.1)	115	0.14
OB (Oligonucleotide/oligosaccharide-binding) fold (b.40)	108	0.34
Ferredoxin-like (d.58)	95	0.15
Adenine nucleotide alpha hydrolase-like (c.26)	92	0.40
Ribonuclease H-like motif (c.55)	79	0.16
NAD(P)-binding Rossmann-fold domains (c.2)	75	0.12
Class II aaRS and biotin synthetases (d.104)	56	0.75
DNA/RNA-binding 3-helical bundle (a.4)	53	0.04
Reductase/isomerase/elongation factor common domain (b.43)	51	0.43
Flavodoxin-like (c.23)	51	0.11

**Table 5:** Over-represented SCOP folds in minimal organisms. Which SCOP folds are most over-represented in minimal organisms, relative to *E. coli*? The first column gives the name and SCOP sccs identifier for folds from SCOP 1.67. The second column gives the total number of domains with each fold among the five organisms. The third column is calculated as the average number of domains among the five minimal organisms studied that were assigned to each fold, divided by the number of domains in *E. coli *assigned to the same fold. 37 other folds also have a ratio of 1.0 and 1 representative in each minimal organism.

**Fold Name**	**Number**	**Ratio**
DNA primase core (e.13)	7	1.4
An anticodon-binding domain of class I aminoacyl-tRNA synthetases (a.97)	6	1.2
Head domain of nucleotide exchange factor GrpE (b.73)	6	1.2
Ribosomal proteins L23 and L15e (d.12)	6	1.2
DNA clamp (d.131)	16	1.1
ValRS/IleRS/LeuRS editing domain (b.51)	15	1.0
S-adenosylmethionine synthetase (d.130)	15	1.0
Dihydrofolate reductases (c.71)	10	1.0
Ribosomal protein L6 (d.141)	10	1.0
beta and beta-prime subunits of DNA dependent RNA- polymerase (e.29)	10	1.0

**Table 6:** SCOP folds in minimal organisms but not *E. coli*. Which SCOP folds are found in minimal organisms, but not *E. coli*? The total number of domains from all five minimal organisms that were assigned to each fold is given in the second column.

**Fold Name**	**Number**
alpha-2-Macroglobulin receptor associated protein (RAP) domain (a.13)	1
STAT-like (a.47)	1
Annexin (a.65)	1
DBL homology domain (DH-domain) (a.87)	1
Non-globular all-alpha subunits of globular proteins (a.137)	1
GatB/YqeY domain (a.182)	2
gamma-Crystallin-like (b.11)	1
SMAD/FHA domain (b.26)	3
Sortase (b.100)	1
C-terminal autoproteolytic domain of nucleoporin nup98 (b.119)	1
Nucleoplasmin-like/VP (viral coat and capsid proteins) (b.121)	2
Hypothetical protein TM1070 (b.123)	1
Hypothetical protein YojF (b.128)	1
Amidase signature (AS) enzymes (c.117)	2
DegV-like (c.119)	2
Urease, gamma-subunit (d.8)	1
Penicillin-binding protein 2x (pbp-2x), c-terminal domain (d.11)	2
MHC antigen-recognition domain (d.19)	1
Thymidylate synthase-complementing protein Thy1 (d.207)	1
Smc hinge domain (d.215)	1
Polo-box domain (d.223)	1
